# Lutetium-177 Radiolabeled Gold Nanoparticles for Prostate Cancer Theranostics

**DOI:** 10.3390/nano16070441

**Published:** 2026-04-04

**Authors:** Adamantia Apostolopoulou, Evangelia-Alexandra Salvanou, Christos Liolios, Stavros Xanthopoulos, Przemysław Koźmiński, Penelope Bouziotis

**Affiliations:** 1 Radiochemical Studies Laboratory, Institute of Nuclear & Radiological Sciences & Technology, Energy & Safety, National Center for Scientific Research “Demokritos”, Agia Paraskevi, 15341 Athens, Greece; a.apostolopoulou@rrp.demokritos.gr (A.A.); salvanou@rrp.demokritos.gr (E.-A.S.); cliolios@pharm.uoa.gr (C.L.); staxan@rrp.demokritos.gr (S.X.); 2 Department of Medicine, National and Kapodistrian University of Athens, Mikras Asias 75, 11527 Athens, Greece; 3 Department of Pharmacy, National and Kapodistrian University of Athens, Panepistimiopolis Zographou, 15771 Athens, Greece; 4 Centre of Radiochemistry and Nuclear Chemistry, Institute of Nuclear Chemistry and Technology, Dorodna 16, 03-195 Warsaw, Poland; p.kozminski@ichtj.waw.pl

**Keywords:** gold nanoparticles, lutetium-177, PSMA, prostate cancer, radiolabeling, MTT assay, spheroids, cell binding, cell internalization

## Abstract

Gold nanoparticles (AuNPs) have been extensively studied in cancer treatment research since they have special physicochemical characteristics such as facile surface functionalization with various chemical groups, low toxicity, favorable biocompatibility, and the ability to passively accumulate in tumors through the enhanced permeability and retention (EPR) effect. Prostate cancer cells exhibit an overexpression of the Prostate-Specific Membrane Antigen (PSMA), which therefore represents an ideal candidate for the development of nanoplatforms targeting PSMA overexpressed on these cells. Lutetium-177 (^177^Lu) is a β-particle emitter with a half-life of 6.7 days. This radionuclide is very promising for the development of theranostic platforms as it emits β^−^ particles, which are suitable for therapy, and γ-photons, capable of SPECT imaging. The combination of ^177^Lu with AuNPs functionalized with PSMA for targeted delivery offers a promising tool for both diagnosis and therapy of prostate cancer. In this study, we focused on the synthesis and in vitro evaluation of PSMA-targeted AuNPs radiolabeled with ^177^Lu. The AuNPs were functionalized with the TADOTAGA chelator, which enables effective radiolabeling with the radiometal, as well as with a PSMA molecule, which comprises the PSMA targeting moiety (vehicle) of the nanoconstruct. Radiolabeling of the functionalized AuNPs with ^177^Lu was fast and robust. Subsequent studies focused on the in vitro stability and cellular interaction with two prostate cancer cell lines with different PSMA expression levels, in both 2D and 3D cell cultures, to assess effective targeting. Results indicate that radiolabeled AuNPs exhibit selective interaction with PSMA-expressing cells and present a stronger in vitro cytotoxic effect when functionalized with the PSMA molecule, confirming their potential as theranostic agents and warranting further investigation in LNCaP tumor-bearing mice.

## 1. Introduction

Over the last few decades, cancer has been the leading cause of death worldwide. More specifically, prostate cancer is the main type of cancer in men, and it has recently been reported that prostate cancer cases will gradually increase from 1.4 million in 2020 to 2.9 million by 2040 [1]. Each tumor presents a unique nature and heterogeneity, which makes this disease extremely complex. Therefore, every patient exhibits a unique pattern of biomarker expression. Consequently, the need for precise and personalized diagnostic and therapeutic applications seems to be essential and warrants further investigation and research. The synergy of nanotechnology and nuclear medicine holds great potential in accomplishing this objective [2,3].

Nanoparticles have been widely explored over the last few decades as very promising candidates for cancer theranostics, as they can be engineered to improve diagnostic and therapeutic outcomes. Their nano-scale size makes them able to passively target tumors via the EPR effect (due to poor lymphatic drainage in growing tumors). Active targeting can be achieved through functionalization of their surface with targeting moieties such as peptides, antibodies, etc. [2]. AuNPs in particular are very attractive nanoplatforms since they offer several advantages, such as a huge variety of sizes and shapes, which could be modified for particular uses. Additionally, AuNPs present unique physicochemical properties such as Localized Surface Plasmon Resonance (LSPR) and fluorescence, excellent biocompatibility, and easy functionalization of their surface with many chemical groups and chelators. Their optical properties enable many therapeutic applications, such as Photothermal and Photodynamic therapy and multimodal imaging [4,5].

The selection of the appropriate radionuclide for the development of effective nanoradiopharmaceuticals is crucial [6]. Lutetium-177 (^177^Lu) is a medium-energy β^−^-emitting radionuclide widely used in targeted radionuclide therapy and theranostic applications. Its half-life of ~6.65 days and emission of β^−^ particles with a maximum energy of ~0.497 MeV make it suitable for treating small- to medium-sized tumors while minimizing damage to surrounding healthy tissue. Additionally, ^177^Lu emits low-energy γ-photons (113 keV and 208 keV), enabling simultaneous Single-Photon Emission Computed Tomography (SPECT) imaging and dosimetry [7]. Clinically validated applications of ^177^Lu include radiopharmaceuticals such as Lutathera and Pluvicto, with demonstrated efficacy in peptide receptor radionuclide therapy (PRRT) and PSMA-targeted therapy, respectively. All the aforementioned characteristics of ^177^Lu, as well as its availability and the ability for stable complexation into nanoparticle-based delivery systems, make it an ideal candidate for nanosized radiopharmaceuticals for cancer treatment [8].

For conjugation of ^177^Lu to nanoparticles, macrocyclic chelators are most commonly employed due to their high thermodynamic stability and kinetic inertness with trivalent lanthanides [9]. Among these, DOTA (1,4,7,10-tetraazacyclododecane-1,4,7,10-tetraacetic acid) and derivatives such as DOTAGA are considered the gold standard, forming highly stable Lu^3+^ complexes that resist transchelation and in vivo dissociation. Acyclic chelators such as diethylenetriaminepentaacetic acid (DTPA), or direct labeling procedures, have also been explored but typically exhibit lower kinetic stability compared to DOTA-based systems. The choice of chelator and conjugation strategy critically influences radiochemical yield and stability, and therefore in vitro and in vivo behavior of the radiocomplexes.

PSMA is a type II transmembrane glycoprotein, also known as glutamate carboxypeptidase II or folate hydrolase I [10,11,12,13,14]. It is an antigen consisting of a 707-amino acid extracellular region, a 24-amino acid transmembrane domain, and a 19-amino acid cytoplasmic fragment [12,13]. In regular prostatic tissue, PSMA is located on the cytoplasmic and apical sides of epithelial cells. During malignant transformation, it is overexpressed, and its large extracellular domain becomes available for ligand binding. Prostate adenocarcinoma can express PSMA up to 100–1000 times higher than those in benign prostate tissue, while normal tissues physiologically present lower levels [11,12,13,14,15,16,17]. Small-molecule PSMA ligands and antibodies can specifically target cancer cells by attaching to the extracellular domain and internalizing into the cell. Clinical guidelines now include the use of radiolabeled PSMA ligands, such as ^68^Ga-PSMA-11 and ^18^F-PSMA-1007, to accurately detect primary and metastatic tumors through PET imaging [10,13,14]. PSMA is also a target for therapy, enabling the delivery of therapeutic radionuclides to cancer cells with high specificity and without harming healthy tissues. Radioligand therapy with β-emitters such as ^177^Lu-PSMA-617 or alpha-emitters such as ^225^Ac-PSMA-617 causes DNA damage and apoptosis of cancer cells [10,14]. The internalization of PSMA is also an advantage that increases the efficacy of these treatments [12,13,14]. In summary, the unique characteristics of PSMA, such as its overexpression in tumors and the ability to be internalized, make it an ideal candidate for targeting prostate cancer for imaging and therapeutic applications.

The combination of nanoparticles and PSMA is an extremely promising approach for targeted diagnosis and therapy of prostate cancer. Functionalization of the surface of nanoparticles with PSMA-targeting molecules can selectively enable binding to prostate cells. The internalization achieved due to the presence of PSMA can enhance intracellular delivery of the radiolabeled nanoparticles, leading to more pronounced accumulation within cancer cells while at the same time reducing damage to the healthy tissues. AuNPs administered via intravenous injection (i.v.) mainly accumulate in reticuloendothelial system (RES) organs (liver and spleen) due to the phenomenon of opsonisation [18,19,20,21,22]. Nanobrachytherapy involves intratumoral (i.v.) administration of the radiolabeled nanostructures, leading to localized accumulation within the tumor mass and reduced off-target toxicity due to minor distribution in other non-targeted organs or healthy tissues. [19,20,23]. Research efforts focusing on the development of PSMA-targeted nanoplatforms have been significantly increased over the last few decades, highlighting the potential of these two approaches (i.v. vs. i.t.) [24,25,26,27,28].

In the present study, we focused on the formation of a PSMA-functionalized gold-based nanoconjugate that could be radiolabeled with the β-emitter ^177^Lu via the DOTA-derivative chelator TADOTAGA. This study is innovative compared to previously reported AuNP–^177^Lu systems by shifting from a passive approach to a more targeted strategy, to demonstrate the role of PSMA-mediated delivery of AuNPs−^177^Lu systems. Moreover, our objective was also to directly compare PSMA-functionalized and non-functionalized ^177^Lu-AuNPs at the cellular level. Based on current information reported in the literature, this direct comparison has not been thoroughly investigated so far. To the best of our knowledge, this is the first time that such a nanoplatform is being reported, as there are multiple nanosystems that have been described in the literature combining AuNPs with ^177^Lu, but none of them with this specific chelator or prostate cancer targeting moiety [29].

## 2. Materials and Methods

### 2.1. Chemical Reagents and Equipment

All reagents and solvents were used without further purification. Acetonitrile (CH_3_CN, >99.5%) and Sodium Hydroxide (NaOH) were purchased from Carlo Erba (Val-de-Reuil, France). Octanol-1 (99%) was purchased from Acros Organics (Geel, Belgium). Hydroclorid acid (TraceSELECT for trace analysis ≥ 37%) was obtained from Honeywell (Michigan, USA). Citric acid was purchased from Merck (Darmstadt, Germany). Glycine (99%) and Dimethyl Sulphoxide (DMSO) were obtained from Panreac (Barcelona, Spain). Water (HPLC grade and Ultra Trace Elemental Analysis Grade), Trisodium citrate (99.5%), and Phosphate-Buffered Saline Tablets were purchased from Fisher Scientific (Leicestershire, UK). Trifluoroacetic acid (TFA, >99%) and N,N′-Diisopropylcarbodiimide (DIC) were purchased from Alfa Aesar (Lancashire, UK). Human serum (H4522-20 mL from human male AB plasma, USA origin, sterile filtered), Albumin bovine serum, Gold Chloride trihydrate (HAuCl_4_∙3H_2_O), Diethylenetriaminepentaacetic acid (≥99%), tetrakis(triphenylphosphine)palladium(0), 4-methyl-piperidine, morpholine, Ethane-1,2-dithiol (EDT), Sodium Diethyldithiocarbamate and Sodium acetate (99.995% trace metals basis) were purchased from Sigma Aldrich (St. Louis, MO, USA). Oxyma pure and 2-Chlorotrityl chloride resin (2-CTC) (100–200 mesh, 1% DVB) were obtained from NovaBiochem (Nottingham, UK), while Dichloromethane (DCM), Diethyl ether, and Fmoc-Lys(Alloc)-OH were obtained from Chem-Lab Analytical (Zedelgem, Belgium). Moreover, Petroleum ether and Dimethylformamide (DMF) were purchased from Lab-scan (Gliwice, Poland). Mass spectroscopy was performed on a Waters Q-Tof Premier Mass spectrometer (Waters Corporation, Milford, MA, USA). The AuNPs were characterized regarding their hydrodynamic diameter and zeta potential (ζ_p_) by dynamic light scattering (DLS, Malvern, Worcestershire, UK). HPLC was performed using a Waters 600 Controller pump and a Waters 996 Photodiode Array detector (Massachusetts, USA), on a semi-prep C18 column (Macherey-Nagel, Duren, Germany). The UV detection wavelength was set simultaneously at 220 and 254 nm for all experiments. The HPLC solvents of analytical grade were filtered through 0.22 mm membrane filters (Millipore, Milford, MA, USA). All samples were centrifuged using Universal Centrifuge Z 326 K (Hermle Labortechnik, Wehingen, Germany). Glass microfiber chromatography paper impregnated with silica gel (ITLC-SG) was purchased from Agilent Technologies (Santa Clara, CA, USA) and was used in the determination of radiolabeling yield during radiolabeling and stability studies, on a radio-TLC scanner (Scan-Ram, LabLogic, Sheffield, UK). Data collection and analysis of radiolabeling were performed with Laura software v. 5.0.4.29. Radioactivity measurements were conducted in a dose calibrator (Capintec, Ramsey, New Jersey). Samples for lipophilicity and in vitro binding studies were measured on a Packard COBRA II Auto-Gamma Counter (Canberra, Meriden, USA). Lutetium-177 was provided as [^177^Lu]LuCl_3_ by ITM Isotope Technologies Munich SE. The cells were observed under an optical microscope (Optica Microscopes, Via Rigla, Italy), while Image Focus software (Version: x64, 1.3.7.27993.20250313) (Εuromex, Arnhem, Netherlands) was used for photographs and spheroid analysis.

The prostate cancer cell lines used (LNCaP and PC3) were acquired from ATCC (LGC Standards, Middlesex, UK). RPMI and DMEM were used as media for the cultures and were purchased from Biowest (Nuaillé, France). The MTT reagent [3-(4,5 –dimethylthiazol-2-yl)-2,5-diphenyltetrazolium bromide] was obtained from Applichem (Darmstadt, Germany). All flasks and well plates used were purchased from TPP (Trasadingen, Switzerland). For the Optical Density Measurements, a LabSystems Multiskan RC Microplate Reader (Thermo Fisher Scientific, Waltham, MA, USA) was used.

### 2.2. Synthesis and Characterization of the PSMA-Targeting Molecule

**Figure 1:**
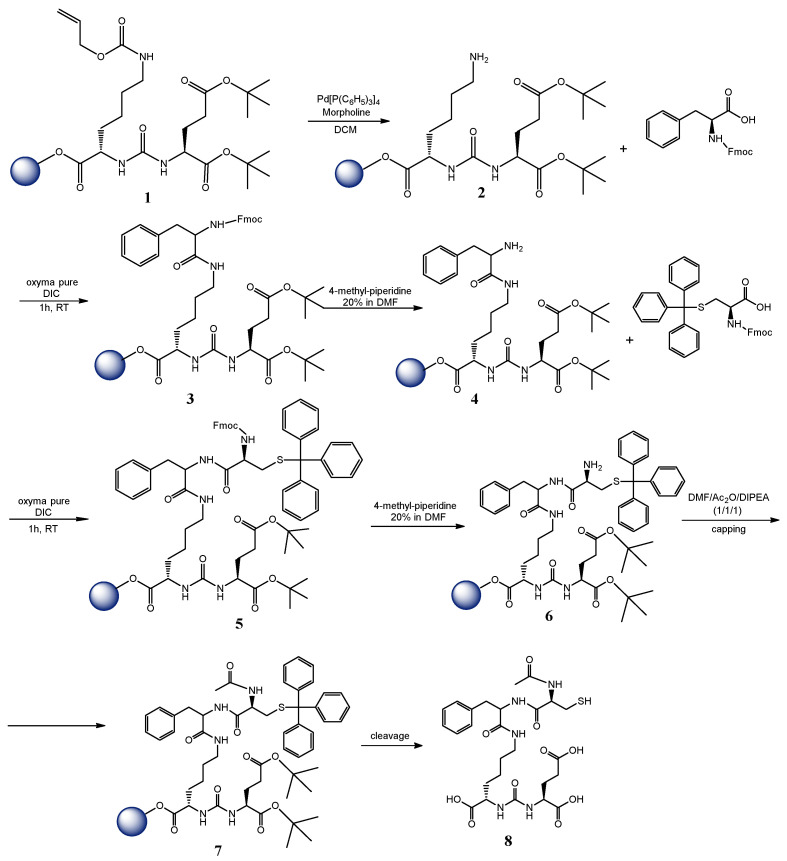
Chemical synthesis of PSMA-targeting molecule **8** using SPPS.

The synthesis of the PSMA-SH derivative (Figure 1) was accomplished by SPPS, according to previously published methods (S.I.) [30,31,32] on a 2-CTC resin. The crude product 8 was cleaved from the resin, purified via a semi-prep RP—HPLC, and identified with ESI-MS (S.I.).

### 2.3. Synthesis and Characterization of the AuNPs

The synthesized AuNPs have been previously described and were synthesized according to the modified Turkevich method [33,34]. Briefly, for the synthesis of AuNPs with a hydrodynamic diameter of 20 nm, 29.54 mg gold(III) chloride trihydrate was dissolved in 100 mL of distilled water and heated under reflux in a round-bottom flask. Next, 83.84 mg trisodium citrate dehydrate was dissolved in 10 mL of distilled water. During boiling, the solution of gold(III) chloride trihydrate was added rapidly, and the mixture was further heated for 15 min. After cooling down the AuNPs solution, the flask was wrapped with aluminum foil and stored at 4–8 °C. A correctly prepared solution of AuNPs with a size of 20 nm has a red wine color. The size and the zeta-potential of the nanoparticles were assessed by Dynamic Light Scattering (DLS).

### 2.4. Functionalization and Characterization of the AuNPs

As a first step, AuNPs were surface-functionalized with a chelator enabling their radiolabeling with the radiometal Lutetium-177 (^177^Lu). The chelating agent used was TADOTAGA (Chematech), which contains a thiol group, facilitating a strong attachment onto the AuNPs surface through the formation of stable Au–S bonds (AuNPs-TADOTAGA). Furthermore, the AuNPs were also modified with the previously synthesized PSMA ligand (Section 2.2), which also contains a thiol group for stable attachment to the AuNP surface (AuNPs-TADOTAGA-PSMA). All samples were prepared according to the same procedure, as follows. Briefly, 50 μg (1 pmol) of AuNPs were mixed with 50 μg (70 nmol) of TADOTAGA, and the mixture was left for 2 h. In the case of PSMA-ligand, 6 μg (10 nmol) were added to the nanoparticles prior to the TADOTAGA, and the mixture was left overnight. Both PSMA and TADOTAGA were dissolved in trace-free water with a final concentration of 1 mg/mL Finally, the samples were centrifuged at 12,000 rpm for 20 min, and after acquiring a clear red pellet (~50 μL), the supernatant was carefully removed. The purified AuNPs were ready for radiolabeling, as described below in Section 2.5.

The percentage of the fractions of TADOTAGA and PSMA bound to the AuNPs was calculated using an analytical RP-HPLC method, by integrating the area under the curve (AUC, UV-VIS detector, 220 nm) of the peaks. We first estimated the AUC for a control sample of both TADOTAGA and PSMA corresponding to the amounts used for functionalization of the AuNPs (100%). Then, the supernatants mentioned above underwent HPLC analysis, and the resulting AUCs were subtracted from the AUC of the control samples.

### 2.5. Radiolabeling of AuNPs with Lutetium-177

After functionalization of the AuNPs and purification by centrifugation, radiolabeling followed as described below. Briefly, in two eppendorf tubes containing ~50 μL of our samples (AuNPs-TADOTAGA and AuNPs-TADOTAGA-PSMA) and 200 μL Sodium Acetate Buffer pH 5.5, 5–20 µL [^177^Lu]LuCl_3_ (15–60 MBq) was added. The mixture was incubated at 70 °C for 40 min. The radiolabeling yield was determined by Instant Thin Layer Chromatography (ITLC) using ITLC-SG glass microfiber chromatography paper and 0.1 M citric acid as the mobile phase. In this solvent system, the free Lu^3+^ migrates to the front (Rf = 0.8–1.0) of the ITLC-SG strip, leaving the radiolabeled complexes at the origin (Rf = 0.0–0.2).

### 2.6. In Vitro Stability Studies

After evaluating the radiolabeling yield, the in vitro stability of the radiolabeled nanoparticles was assessed for up to 15 d at Room Temperature (RT) and in Phosphate-Buffered Saline pH 7.4 (PBS, 0.01 M, ratio 1:10). Furthermore, for the same period, the stability of the radiolabeled samples was evaluated in the presence of human serum (1:10) and DTPA 0.01 M (1:1) at 37 °C to evaluate their potential in vivo stability. The percentage of radiochemical stability was determined by ITLC-SG glass microfiber chromatography paper using Citric Acid 0.1 M as the mobile phase.

### 2.7. Lipophilicity Studies

The lipophilicity of the radiolabeled nanoparticles was determined by calculating the partition coefficient (P) with the shake-flask method. In short, 30–70 KBq of each of the radiolabeled nanoparticles was added in a centrifuge tube containing 2 mL of 1-octanol (organic phase) and 2 mL PBS (0.01 M, pH 7.4). After vortexing for 1 min, the radioactivity of the aliquots (200 μL) of each phase was counted in a gamma counter. The following equation was used to calculate the partition coefficient. LogP was used to express the results. The procedure was performed in triplicate [35].

$$P=\frac{\frac{\mathrm{counts}}{\mathrm{mL}}on\;the\;1-octanol\;phase}{\mathrm{counts}/\mathrm{mL}\;on\;the\;PBS\;phase}$$

### 2.8. Cytotoxicity Studies

Cell studies were performed on two different prostate cancer cell lines, namely LNCaP and PC3. The LNCaP cells are androgen-sensitive and naturally express PSMA. On the contrary, the PC3 cells do not express PSMA and were used as the control cell line. For LNCaP cells, the medium used was RPMI, while PC3 cells were cultured in DMEM.

#### 2.8.1. Cytotoxicity Studies on 2D Cell Lines

In vitro cytotoxicity against the PC3 and LNCaP cell lines was evaluated for up to 72 h, using the 3-(4,5-dimethylthiazol-2-yl)-2,5-diphenyltetrazolium bromide (MTT) colorimetric assay. In short, cells were seeded in 96-well plates in different numbers according to the respective incubation times (10 × 10^3^, 8 × 10^3^, and 6 × 10^3^ cells/well for the 24, 48, and 72 h, respectively, for PC3 cells, and 15 × 10^3^, 12 × 10^3^, and 10 × 10^3^ cells/well for the 24, 48, and 72 h for LNCaP cells). Cells remained overnight at 37 °C in a 5% CO_2_ incubator. The concentrations of radiolabeled AuNPs ranged from 0.125 to 4 MBq/mL, which corresponds to 1.5625–50 µg/mL of AuNPs. The final volume in each well was 100 µL at all examined concentrations. The medium was then removed and replaced with 100 µL of MTT solution (1 mg/mL). After 4 h incubation, the medium was removed, the formed formazan crystals were solubilized in 100 µL of DMSO, and absorbance was recorded at 560 nm. The results were expressed as % cell viability = (mean optical density (OD) of treated cells/mean OD of untreated cells) × 100. Each assay was repeated at least three times.

#### 2.8.2. Cytotoxicity Studies on 3D Cell Spheroids

Prostate cancer LNCaP and PC3 cells were added to a 96-well U-bottom microplate, Nuclon Sphera-Treated (Thermo Scientific, Tokyo, Japan), and cultured for five days (~2000 cells/well). Thereafter, [^177^Lu]Lu-AuNPs-TADOTAGA and [^177^Lu]Lu-AuNPs-TADOTAGA-PSMA were added. In parallel, spheroids in a control group were monitored and allowed to grow without the addition of the radioactive compounds. Treated and control spheroids were analyzed over a 15-day period, with medium renewal every 5 days. In this assay, cells were treated with 50 µg_AuNPs_/mL and radioactivity corresponding to 20 MBq/mL. The Relative Surface Area (RSA) was monitored for up to 15 days and was calculated by dividing the spheroid area at each measurement by the initial spheroid area at the initiation of the experiment (day 0). The cells were observed under an optical microscope while Image Focus software was used for photographs and spheroid analysis [36].

### 2.9. In Vitro Binding Studies

One day before the experiment, LNCaP and PC3 cells (2 × 10^5^ and 1.5 × 10^5^ cells/well, respectively) were seeded in 24-well plates and stored in a cell incubator at 37 °C under a 5% CO_2_ atmosphere. The next day, the cells were washed with 1 mL PBS, and a series of different concentrations of [^177^Lu]Lu-AuNPs-TADOTAGA and [^177^Lu]Lu-AuNPs-TADOTAGA-PSMA were added, followed by 1 h incubation. The medium used (RPMI or DMEM) was enriched with 1% bovine serum albumin (BSA). The medium was then collected, and the cells were gently washed with PBS. Finally, 1 M NaOH was added, and the fraction with cells was collected into separate tubes. The activity of the collected fractions was measured using a Packard COBRA II Auto-Gamma Counter (Canberra, USA). To calculate the specific binding, the difference between total binding and nonspecific binding was determined (PSMA receptors were blocked with a 100-molar excess of 2-(Phosphonomethyl)pentanedioic acid (2-PMPA) [36,37,38].

### 2.10. Internalization Studies

The internalization assay was performed on LNCaP cells. The percentage of the internalized [^177^Lu]Lu-AuNPs-TADOTAGA-PSMA and the percentage attached to the cell membrane were evaluated with the following procedures. Cell preparation for the experiment was identical to the binding experiment (Section 2.9). After removing the medium, 100 pM nanoconjugate in 1 mL medium was added to each well (24-well plates) and the cells were incubated for 1, 2, 4, and 24 h at 37 °C. The incubation medium was removed, and the cells were washed with PBS (1 mL). In the next step, the cells were treated with ice-cold 0.05 M glycine·HCl pH = 2.8 (1 mL) to determine the radiolabeled complex that is bound to the cell membrane but not internalized. Finally, 1 M NaOH was added to the cells for 10 min to collect the internalized fraction. All collected fractions were measured using a Packard COBRA II Auto-Gamma Counter (Canberra, USA) [36,37,38].

## 3. Results and Discussion

### 3.1. Synthesis and Characterization of the PSMA-Targeting Molecule

The PSMA targeting molecule was synthesized with SPPS according to previously published methods [30,31,32]. The pure product **8** was analyzed by RP-HPLC applying a gradient system from 10% B to 80% B at 13 min and from 80% B at 15 min to 10% B at 17 min, where solvent A was H_2_O/0.1% TFA and solvent B was ACN/0.1% TFA, at a flow rate of 2 mL/min using a semi-prep C18 column showing a purity of 98% (Figure S2), and subsequently identified with ESI-MS (Figure S1). The purified fraction was lyophilized to a white powder to be later used for binding to the AuNPs through the thiol group.

### 3.2. Synthesis and Characterization of the AuNPs

**Figure 2:**
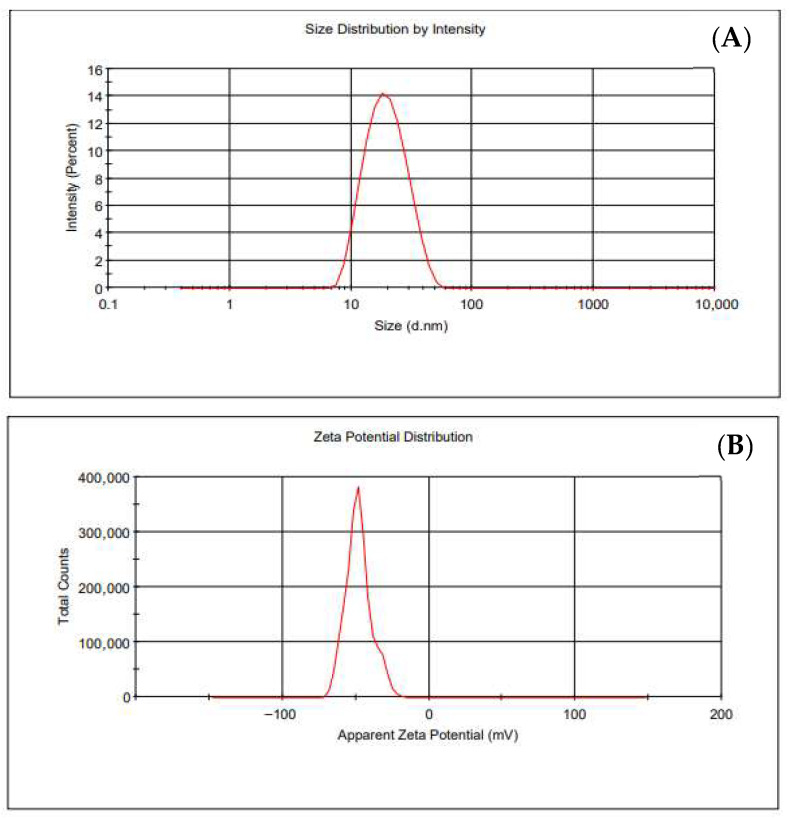
Particle size distribution by intensity (**A**) and Zeta Potential distribution (**B**) as determined by DLS.

The synthesized AuNPs had an average hydrodynamic diameter of 20 nm, as indicated by the DLS measurements. The results of the size and zeta potential of the nanoparticles are presented in Table 1 and Figure 2. The negative zeta potential indicates that the synthesized AuNPs are stable and there was no tendency for the particles to aggregate.

### 3.3. Functionalization and Characterization of the AuNPs

The functionalized AuNPs were also characterized by DLS. The results are summarized in Table 2 regarding their hydrodynamic diameter and zeta potential.

The observed increased hydrodynamic diameters confirm the successful surface functionalization. The difference between AuNPs-TADOTAGA and AuNPs-TADOTAGA-PSMA may be attributed to the competitive binding of the chelator and the PSMA via their thiol groups. In the case of targeted AuNPs, the PSMA molecule is added prior to the chelator and covers several binding sites on the surface of the AuNPs, thereby reducing the available sites for TADOTAGA. On the contrary, the absence of PSMA provides unlimited access for the chelator to bind to the AuNPs. This behavior leads to higher surface coverage and a larger hydrodynamic diameter [39]. Colloidal stability is a crucial issue, especially for NPs used for biomedical applications. Therefore, TADOTAGA and PSMA that are covalently linked to the surface of the NPs by Au–S bonds confer a largely negative zeta potential value to the NPs, thus preventing their aggregation in solution [40].

The percentage of surface functionalization of the AuNPs with the TADOTAGA chelator and PSMA targeting moiety was estimated by HPLC. The percentage of bound PSMA was 74.1 ± 3.65%, while the percentage of bound TADOTAGA was 24.3 ± 5.32%, regardless of previous functionalization of the AuNPs with PSMA.

### 3.4. Radiolabeling of AuNPs with Lutetium-177

The radiolabeling yield of AuNPs with ^177^Lu was found to be 98.55 ± 0.63% for [^177^Lu]Lu-AuNPs-TADOTAGA and 97.15 ± 1.69% for [^177^Lu]Lu-AuNPs-TADOTAGA-PSMA, respectively, as determined by ITLC-SG glass microfiber chromatography. These results are consistent with, and comparable to, previously reported ^177^Lu-labeled nanocomplexes employing macrocyclic chelators such as DOTA or its derivatives, where incorporation efficiencies are typically > 90% under similar radiolabeling conditions [9]. The aim of the functionalization was to provide a chelator for the stable coordination and robust radiolabeling of AuNPs with the radiometal ^177^Lu, as well as a PSMA moiety for selective targeting. After functionalization, strong Au-S bonds were formed between the AuNPs, the chelator, and PSMA. Thiol groups are considered to be the most important type of molecule to stabilize any size of AuNPs [21,41].

### 3.5. In Vitro Stability Studies

**Figure 3:**
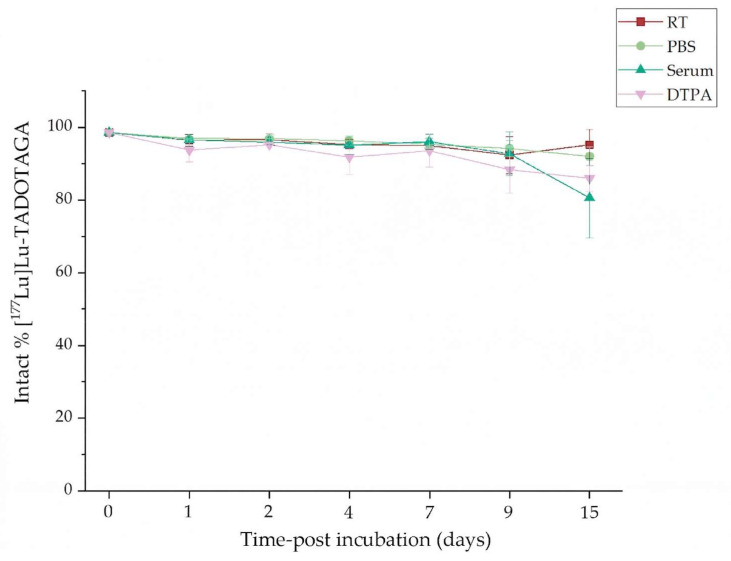
Radiochemical stability data of [^177^Lu]Lu-AuNPs-TADOTAGA at RT (Bench stability), in the presence of human serum, PBS and DTPA at 37 °C for up to 15 days (x axis not in scale).

**Figure 4:**
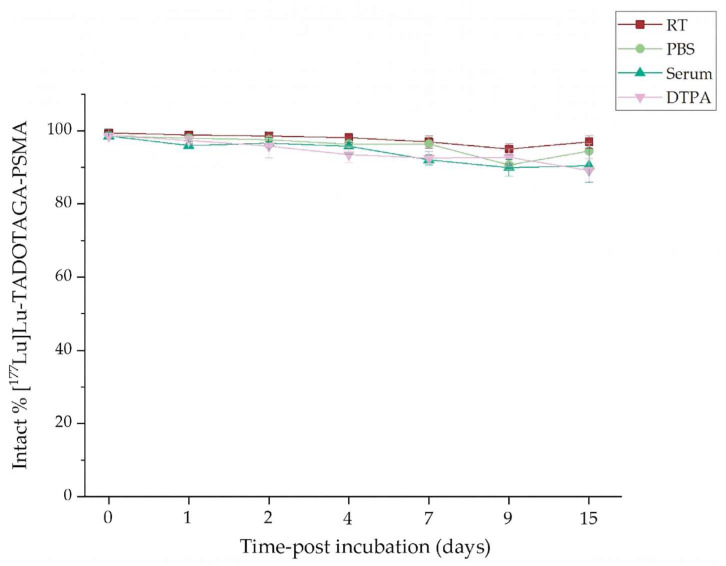
Radiochemical stability data of [^177^Lu]Lu-AuNPs-TADOTAGA-PSMA at RT (Bench stability), in the presence of human serum, PBS, and DTPA at 37 °C for up to 15 days (x axis not in scale).

Regarding the in vitro stability studies, both types of radiolabeled nanoparticles exhibited unique radiochemical stability under all examined conditions (at RT, in the presence of PBS, human serum, and DTPA at 37 °C) for up to 15 days, and the results are summarized in the figures below (Figure 3 and Figure 4).

More specifically, the [^177^Lu]Lu-AuNPs-TADOTAGA demonstrated excellent stability at RT and in PBS (95.22 ± 4.16%, and 92.02 ± 2.60% on day 15, respectively). On the other hand, a slight decrease in stability was observed in the presence of human serum (from 96.54 ± 1.10% on day 1 to ~80% on day 15, remaining >90% on day 9). Regarding the DTPA challenge, a satisfactory resistance to transchelation was observed, where we observed >85% retention of intact radiolabeled complex even at the final time point.

Likewise, the [^177^Lu]Lu-AuNPs-TADOTAGA-PSMA also presented high in vitro stability at all tested conditions. Stability at RT and in PBS remained >90% even after 15 days. Ιn human serum, the intact radiolabeled fraction remained above 95% at early time points (day 1 to day 4) and gradually decreased over time, reaching 86.92 ± 3.59% on day 15. Finally, in the presence of DTPA, a time-dependent decline was observed, leading to a final stability of 85.96 ± 0.67% at the final time point (day 15).

Similar results showing enhanced radiolabeling yield and stability using the TADOTAGA chelator for the conjugation of Terbium-161 in Au-based nanostructures have been previously reported by Salvanou et al. [19]. Our results indicate enhanced stability of the radiocomplex in serum when compared to ^177^Lu-labeled AuNPs modified with polyethylene glycol (PEG) chains and DOTA chelators linked to panitumumab (OPSS-PEG-DOTA−^177^Lu), which were reported to lose ~10% of the radiometal after a 5 d incubation in human serum [42].

The results reveal that both nanoparticle complexes have strong radiochemical stability under all circumstances. However, PSMA-functionalized nanoparticles presented slightly reduced stability at final timepoints, particularly at RT and in PBS. This behavior can be attributed to competitive interactions between the PSMA moiety and the TADOTAGA chelator for available binding sites on the surface of the nanoparticle. Despite these differences, their overall stability profiles indicate that they are suitable for additional in vitro evaluation and in vivo studies.

### 3.6. Lipophilicity Studies

The partition coefficients (P) were determined using the shake-flask method as described above and are expressed as logP. Both types of radiolabeled nanoparticles exhibited hydrophilic behavior (logP < 0). More specifically, when compared, the PSMA-targeted AuNPs presented higher values of logP (logP_AuNPs-TADOTAGA-PSMA_ > logP_AuNPs-TADOTAGA_). This slightly lipophilic behavior may be attributed to the presence of an aromatic ring in the chemical structure of PSMA. The results are presented in Table 3.

### 3.7. Cytotoxicity Studies

#### 3.7.1. Cytotoxicity Studies on 2D Cell Lines

**Figure 5:**
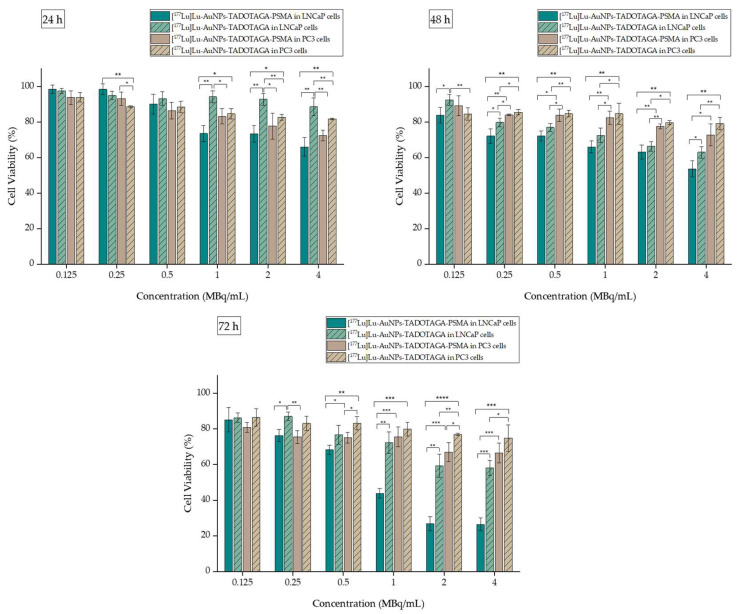
Cell viability assessed by MTT assay of [^177^Lu]Lu-AuNPs-TADOTAGA and [^177^Lu]Lu-AuNPs-TADOTAGA-PSMA against the LNCaP and PC3 cell line for 24, 48, and 72 h. (* *p* < 0.05, ** *p* < 0.01, *** *p* < 0.001, **** *p* < 0.0001; absence of asterisks indicates a non-significant statistical difference). Values represent the mean ± SD (n = 3).

The results of the cell toxicity studies after incubation of [^177^Lu]Lu-AuNPs-TADOTAGA and [^177^Lu]Lu-AuNPs-TADOTAGA-PSMA for up to 72 h are summarized in Figure 5. As indicated, the incubation of [^177^Lu]Lu-AuNPs-TADOTAGA-PSMA on LNCaP cells causes a strong reduction in cell viability starting even at 24 h on higher activity concentrations (1, 2, and 4 MBq/mL), reaching ~26.34 ± 3.66% at 72 h at 4 MBq/mL. At the same time, [^177^Lu]Lu-AuNPs-TADOTAGA respectively reach a maximum decrease in cell viability of 58.16 ± 4.17% in the same cell line. The difference between the two radiolabeled complexes is significant at almost all examined timepoints and concentrations. This result confirms that the presence of the PSMA moiety significantly enhances the cytotoxic behavior due to the effective targeting of PSMA on LNCaP cells.

In contrast, PC3 cells with little to no PSMA expression presented significantly higher cell viability across all tested activities and timepoints. At the highest activities and longer incubation times examined, a more moderate cytotoxicity was observed, which remains significantly lower than that observed in LNCaP cells. More specifically, the maximum decrease in cell viability is 66.43 ± 5.54% for [^177^Lu]Lu-AuNPs-TADOTAGA-PSMA and 74.91 ± 7.41% for [^177^Lu]Lu-AuNPs-TADOTAGA at 72 h at 4 MBq/mL. Moreover, incubation with targeted AuNPs does not cause a notable difference in cytotoxicity when compared to non-targeted AuNPs, indicating the lack of PSMA expression in this cell line. To summarize, these findings confirm the high specificity of targeted AuNPs, highlighting that the presence of the PSMA moiety is crucial for effective prostate cancer therapy.

**Figure 6:**
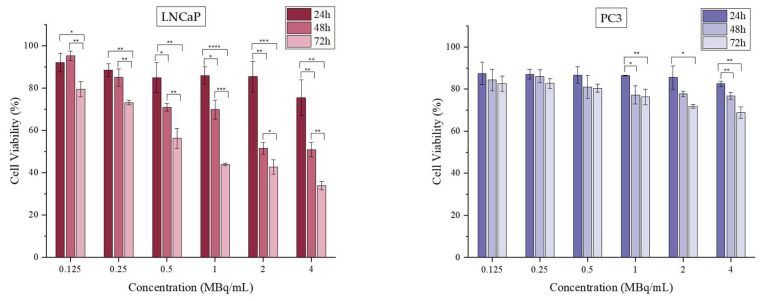
Cell viability assessed by an MTT assay of [^177^Lu]LuCl_3_ against the LNCaP and PC3 cell line for up to 72 h. (* *p* < 0.05, ** *p* < 0.01, *** *p* < 0.001, **** *p* < 0.0001; absence of asterisks indicates a non-significant statistical difference). Values represent the mean ± SD (n = 3).

Overall, it is worth noting that both radiolabeled nanocomplexes present enhanced cytotoxicity in LNCaP cells even in the absence of the PSMA molecule. Thus, it was very interesting to also examine the effect of ^177^LuCl_3_ at the same activity range on both cell lines. The results are summarized in Figure 6.

The MTT assay demonstrated a cytotoxic effect of [^177^Lu]LuCl_3_ in LNCaP cells, while PC3 cells presented a more moderate effect at all tested conditions. At prolonged incubation times (48 and 72 h), LNCaP cell viability was reduced, reaching 33.83 ± 1.98% at 72 h and at the highest examined activity (4 MBq/mL). In contrast, PC3 cells reached only 68.84 ± 2.74% at the same activity and timepoint. The different effect of radioactivity of [^177^Lu]LuCl_3_ up to 5 MBq/mL and at the same timepoints has been reported before [20]. The evaluation was performed in five different breast cancer cell lines (4T1, MDA-MB-231, M165, MCF7, and SKBR3) and verified that the precursor [^177^Lu]LuCl_3_ can have a time- and dose-dependent effect in some cancer cell lines, similar to the difference indicated in our work between LNCaP and PC3 cells. Our results are also in accordance with the results mentioned by Chen M.-W. et al., who also reported the different cell viability effects of ^177^Lu and ^177^Lu-PSMA-NARI-56 in LNCaP and PC3 cell lines [43].

These findings further support the previous results obtained after performing MTT assay on targeted and non-targeted AuNPs, where higher cytotoxicity was observed in LNCaP cells. More importantly, it is demonstrated that the pronounced sensitivity of LNCaP cells to [^177^Lu]LuCl_3_ is not entirely caused by the presence of the PSMA moiety, but it is definitely enhanced.

#### 3.7.2. Cytotoxicity Studies on 3D Cell Lines

2D cell cultures have been widely used over the past decades as a useful tool for the in vitro evaluation of anticancer drugs due to their simplicity and low cost. Unfortunately, 2D cell models are not always able to resemble cellular interactions that take place in vivo. On the other hand, 3D mono-multicellular spheroids can significantly reduce the gap between in vivo and 2D cell cultures, as they bear close similarity to pathophysiological tissue organization and complexity [44,45,46,47,48,49].

**Figure 7:**
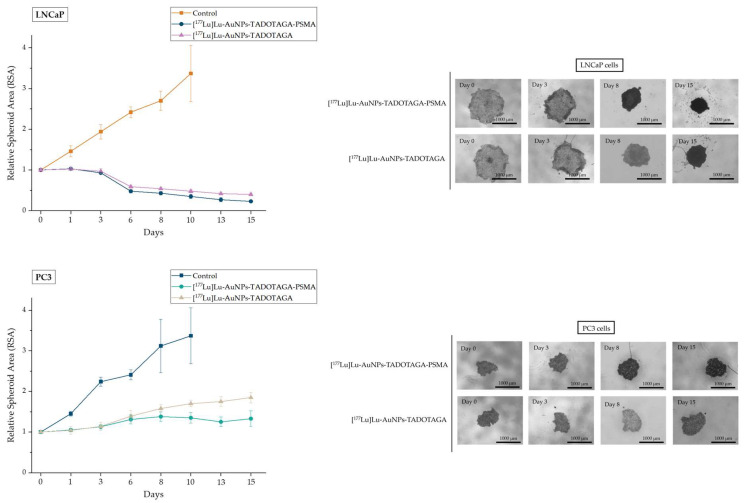
LNCaP and PC3 spheroids treated with [^177^Lu]Lu-AuNPs-TADOTAGA-PSMA and [^177^Lu]Lu-AuNPs-TADOTAGA or non-treated. Data represent the mean ± SD (n = 3).

Microscopic images of the spheroids treated with the synthesized compounds are presented in Figure 7 The diversity of the spheroid morphology between the two different cancer cell lines at day 0 is expected, as it varies depending on the cell line [50].

As shown in Figure 7, the two prostate cancer cell lines exhibited different responses. By day 15, the RSA of LNCaP spheroids treated with [^177^Lu]Lu-AuNPs-TADOTAGA-PSMA had significantly decreased to an RSA of 0.23. A strong but smaller decrease was also observed in LNCaP spheroids treated with [^177^Lu]Lu-AuNPs-TADOTAGA (RSA was reduced to 0.5 by day 15). On the contrary, PSMA-treated PC3 spheroids presented a high increase in size compared to their initial area, but their growth rate was clearly slower when compared to PC3 spheroids treated with [^177^Lu]Lu-AuNPs-TADOTAGA, without PSMA. This behavior is consistent with the low-level expression of PSMA receptors in PC3 cells, which, despite being used as a negative control, may still partially respond to the treatment [51,52,53]. Furthermore, in this assay, the cellular architecture of the spheroids plays a significant role in the evaluation of the imposed effect [54]. This means that although the reduction in the tumor spheroid area is one of the most definite parameters, the observed irregular or uneven shape, spheroid compactness, and detachment of cells from the surface layer or edges are also important and reliable features that should be examined when determining the effectiveness of a treatment. Taking this into account, the morphological alterations of the [^177^Lu]Lu-AuNPs-TADOTAGA-PSMA treated LNCaP cells show a clear image of a frayed spheroid along with dissociated cells and cell debris, something that is not observed in the other cases. Moreover, the untreated LNCaP and PC3 spheroids used as control groups increased their initial area, leading to RSAs of 3.37 and 3.12, respectively, on day 15. A statistically significant difference was observed between the control groups and the treated spheroids in both cell lines for all timepoints. Furthermore, spheroids treated with PSMA-functionalized AuNPs showed a significantly stronger decrease in RSA when compared to their non-PSMA counterparts (Figures S3 and S4, SI). Overall, these results are in accordance with the cytotoxicity experiments using the MTT assay described in Section 3.7.1.

Our cytotoxicity results, particularly those derived from the spheroid assay, offer preliminary information on the in vivo behavior of the potential nanoplatforms. Since spheroids better mimic the tumor environment, these findings encourage a first insight into the next phase of the study, where in vivo experiments (biodistribution and therapeutic efficacy studies) will be carried out. Regarding future studies, experience from our previous work [18,19,20,23] leads us to proceed with studies using the ^177^Lu-AuNPs-TADOTAGA-PSMA as a nanobrachytherapy agent after intratumoral (i.t.) administration.

The clearance pathways of intravenously administered nanoparticles have been extensively reported and follow a similar pattern, showing high accumulation in the liver and spleen due to the phenomenon of opsonisation. In order to avoid RES retention, our group focuses on local (i.t.) administration of nanoparticles, which leads to longer tumor retention, resulting in a more pronounced therapeutic effect while at the same time reducing off-target toxicity, thus sparing neighboring healthy tissues. A tumor-localized nanobrachytherapy agent will provide a longer therapeutic window, which may eventually lead to clinical application in cases of prostate cancer.

### 3.8. In Vitro Binding Studies

**Figure 8:**
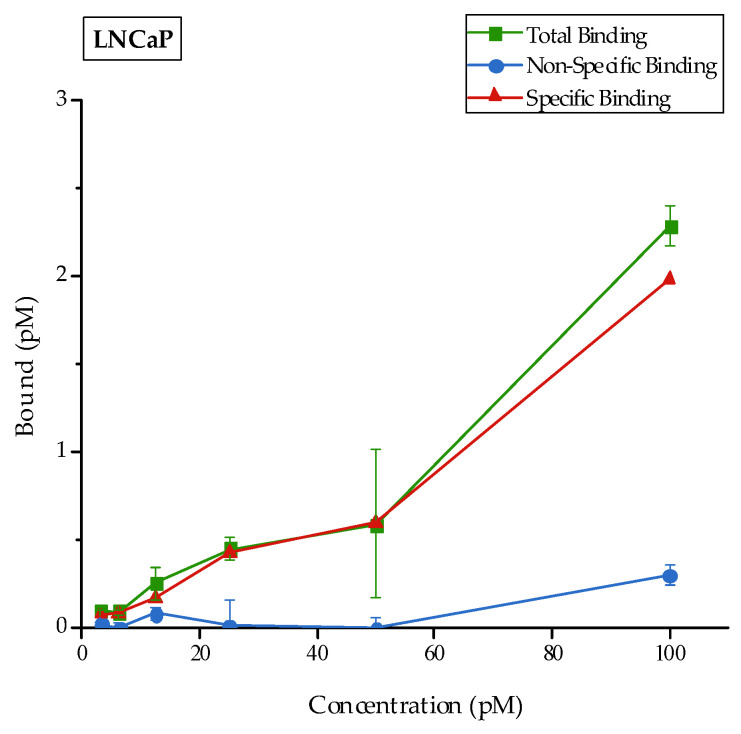
Binding studies of [^177^Lu]Lu-AuNPs-TADOTAGA-PSMA on LNCaP cells and determination of specific binding by blocking with a 100-fold molar excess of 2-PMPA. Data represent the mean ± SD (n = 3).

**Figure 9:**
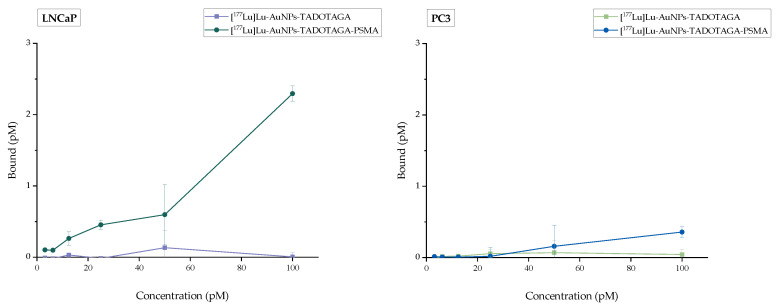
Binding studies of [^177^Lu]Lu-AuNPs-TADOTAGA-PSMA and [^177^Lu]Lu-AuNPs-TADOTAGA on LNCaP and PC3 cells after incubation for 1 h at 37 °C. Data represent the mean ± SD (n = 3).

In vitro binding experiments were carried out by evaluating the receptor binding affinity of [^177^Lu]Lu-AuNPs-TADOTAGA-PSMA in the LNCaP and PC3 prostate cancer cell lines. Blocking studies were additionally conducted to assess the specific activity of the receptor. The results are presented in Figure 8. The green line represents the total activity (sum of specific and nonspecific binding) while the red line corresponds to the specific attachment of the receptor to LNCaP cells. Finally, the blue line indicates the nonspecific binding after blocking the receptors with a 100-fold molar excess of 2-PMPA. Moreover, [^177^Lu]Lu-AuNPs-TADOTAGA were also evaluated in LNCaP and PC3 cells, as additional negative controls. The results are presented in Figure 9 and clearly indicate a lack of binding in both prostate cancer cells lines due to the absence of the PSMA-targeting vector. The radiolabeled complex [^177^Lu]Lu-AuNPs-TADOTAGA-PSMA was also examined in PC3 cells (Figure 9), demonstrating a very small but notable binding when compared to the binding attributed to the AuNPs lacking the PSMA targeting vector. As mentioned above, this behavior may be attributed to the presence of a very small number of PSMA receptors in PC3 cells, despite their widespread use as a negative control [55]. As observed from the results, the difference in binding between LNCaP and PC3 cells remains significant. Overall, it is clearly demonstrated that [^177^Lu]Lu-AuNPs-TADOTAGA-PSMA binds specifically to PSMA receptors expressed on LNCaP cells with high affinity.

### 3.9. Internalization Studies

**Figure 10:**
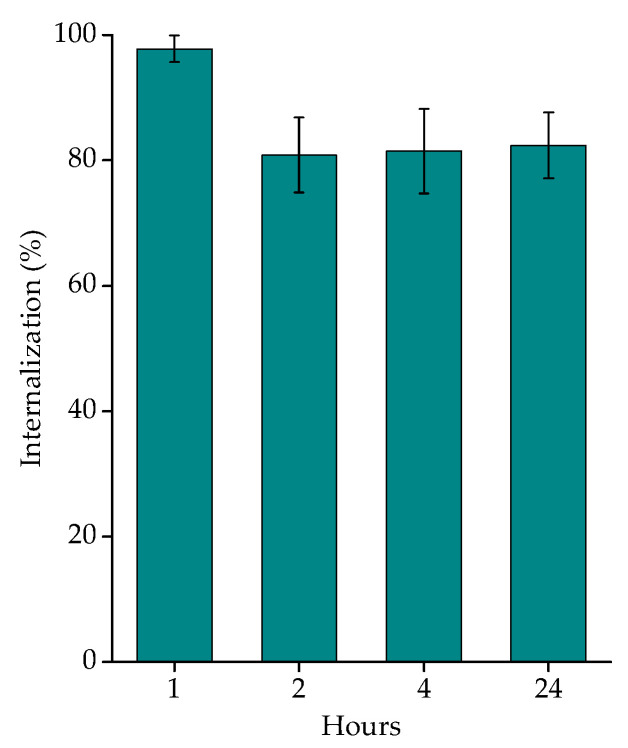
Internalization results of [^177^Lu]Lu-AuNPs-TADOTAGA-PSMA obtained for LNCaP cells after incubation for 1, 2, 4, and 24 h at 37 °C. Data represent the mean ± SD (n = 3).

The internalization of [^177^Lu]Lu-AuNPs-TADOTAGA-PSMA in LNCaP cells was investigated at four time points: 1, 2, 4, and 24 h. The internalization of the PSMA motif is induced via clathrin-dependent endocytosis in LNCaP cells [56,57,58]. Due to the lack of binding, no internalization study was carried out on PC3 cells. The results of internalized [^177^Lu]Lu-AuNPs-TADOTAGA-PSMA at LNCaP cells are presented in Figure 10, with a maximum percentage observed at 1 h. As shown in the figure below, more than 97% (97.80 ± 2.14%) of the radiolabeled complex was internalized after 1 h and remained at high levels for up to 24 h (~80%).

## 4. Conclusions

The AuNPs investigated in the present study were functionalized with a TADOTAGA chelator and a PSMA targeting moiety by forming very strong Au–S bonds. Radiolabeling with the therapeutic radioisotope Lutetium-177 (^177^Lu) was accomplished with yields > 95%. The radiolabeled nanoconstructs also presented unique in vitro stability under all conditions examined for up to 15 days post-radiolabeling. The in vitro cytotoxic profile of [^177^Lu]Lu-AuNPs-TADOTAGA and [^177^Lu]Lu-AuNPs-TADOTAGA-PSMA was investigated against two prostate cancer cell lines with different expressions of PSMA. The MTT assay revealed a pronounced cytotoxic effect in LNCaP cells, thus confirming the effective targeting and therapeutic potential of the PSMA-functionalized AuNPs, in contrast to PC3 cells, which indicated minimal changes in cell viability. The radiolabeled nanoparticles were also examined in 3D LNCaP and PC3 cell spheroids, showing no significant differences in PSMA-negative PC3 spheroids, while PSMA-expressing LNCaP spheroids exhibited a profound effect in spheroid area, particularly after incubation with PSMA-targeted radiolabeled nanoparticles. Finally, the in vitro binding experiments revealed significantly higher binding of PSMA-targeted nanoparticles in LNCaP cells when compared to non-targeted AuNPs or against PC3 cells. In addition, internalization studies indicated that more than 97% of the radiolabeled complex [^177^Lu]Lu-AuNPs-TADOTAGA-PSMA was internalized after just 1 h and remained internalized at high levels for up to 24 h. Based on these preliminary results, both nanocomplexes should be further investigated as nanobrachytherapy agents in LNCaP tumor-bearing mice in order to examine the specific tumor accumulation and therapeutic potential of [^177^Lu]Lu-AuNPs-TADOTAGA-PSMA in vivo.

## Figures and Tables

**Table 1:** Hydrodynamic diameter and zeta potential of bare AuNPs.

Hydrodynamic Diameter (nm)	Zeta Potential (mV)
20.41 ± 2.05	−43.94 ± 2.27

**Table 2:** Hydrodynamic diameter and zeta potential of functionalized AuNPs.

	Hydrodynamic Diameter (nm)	Zeta Potential (mV)
AuNPs-TADOTAGA	25.5 ± 0.4	−37.5 ± 0.7
AuNPs-TADOTAGA-PSMA	22.4 ± 0.7	−27.9 ± 0.7

**Table 3:** Partition Coefficients of radiolabeled nanoparticles; [^177^Lu]Lu-AuNPs-TADOTAGA and [^177^Lu]Lu-AuNPs-TADOTAGA-PSMA.

	logP
[^177^Lu]Lu-AuNPs-TADOTAGA	−1.90 ± 0.80
[^177^Lu]Lu-AuNPs-TADOTAGA-PSMA	−1.44 ± 0.88

## Data Availability

The data presented in this study are available on request from the corresponding author. The data are not publicly available due to IP issues.

## Supplementary Materials

The following supporting information can be downloaded at https://www.mdpi.com/article/10.3390/nano16070441/s1. Figure S1. HPLC graph representing the pure synthesized PSMA-SH on the UV (254 nm) applying a gradient system from 10% B to 80% B at 13 min and from 80% B at 15 min to 10% B at 17 min, where solvent A was H_2_O/0.1% TFA and solvent B was ACN/0.1% TFA, at a flow rate of 2 mL/min using a semi-prep C18 column. Figure S2. Mass Spectrometry graph for the synthesized product PSMA-SH, MW: 611.6670, m/z (M+H) experimental: 612.0866, m/z (M+2H), 613.1438 (60%). Table S1. Radiochemical yield and stability data of [^177^Lu]Lu-AuNPs-TADOTAGA at RT (Bench stability), in the presence of human serum, PBS and DTPA for up to 15 days. Table S2. Radiochemical yield and stability data of [^177^Lu]Lu-AuNPs-TADOTAGA-PSMA at RT (Bench stability), in the presence of human serum, PBS and DTPA for up to 15 days. Figure S3. LNCaP spheroids treated with [^177^Lu]Lu-AuNPs-TADOTAGA-PSMA and [^177^Lu]Lu-AuNPs-TADOTAGA or non-treated (Control group). (* *p* < 0.05, ** *p* < 0.01, *** *p* < 0.001, 98 **** *p* < 0.0001; absence of asterisks indicates a non-significant statistical difference). Values represent the mean ± SD (n = 3). Figure S4. PC3 spheroids treated with [^177^Lu]Lu-AuNPs-TADOTAGA-PSMA and [^177^Lu]Lu-AuNPs-TADOTAGA or non-treated (Control group). (* *p* < 0.05, ** *p* < 0.01, *** *p* < 0.001, **** *p* < 0.0001; absence of asterisks indicates a non-significant statistical difference). Values represent the mean ± SD (n = 3).

## Abbreviations

The following abbreviations are used in this manuscript:

AuNPs	Gold Nanoparticles
BSA	Bovine serum albumine
DCM	Dichloromethane
DIC	N,N′-Diisopropylcarbodiimide
DIPEA	N,N-Diisopropylethylamine
DLS	Dynamic Light Scattering
DMSO	Dimethyl Sulfoxide
DOTA	1,4,7,10-tetraazacyclododecane-1,4,7,10-tetraacetic acid
DTPA	Diethylenetriaminepentaacetic acid
EPR	Enhanced Permeability and Retention
HPLC	High Performance Liquid Chromatography
ITLC	Instant Thin Layer Chromatography
LSPR	Localized Surface Plasmon Resonance
MS	Mass Spectrometry
PBS	Phosphate-Buffered Saline
PEG	Polyethylene glycol
2-PMPA	2 Phosphonomethyl pentanedioic acid
PSMA	Prostate-Specific Membrane Antigen
RES	Reticuloendothelial System
RSA	Relative Surface Area
RT	Room Temperature
SPECT	Single-photon emission computed tomography
TADOTAGA	2,2′,2”-(10-(4-((2-(5-(1,2-dithiolan-3-yl)pentanamido)ethyl)amino)-1-carboxy-4- oxobutyl)-1,4,7,10-tetraazacyclododecane-1,4,7-triyl)triacetic acid
TFA	Trifluoroacetic acid

## References

[1] James N.D., Tannock I., N’Dow J., Feng F., Gillessen S., Ali S.A., Trujillo B., Al-Lazikani B., Attard G., Bray F. (2024). The Lancet Commission on Prostate Cancer: Planning for the Surge in Cases. Lancet.

[2] Silva F., Cabral Campello M.P., Paulo A. (2020). Radiolabeled Gold Nanoparticles for Imaging and Therapy of Cancer. Materials.

[3] Jeon J. (2019). Review of Therapeutic Applications of Radiolabeled Functional Nanomaterials. Int. J. Mol. Sci..

[4] Singh R., Mishra A.K., Bhushan B., Rawat H., Kumar V. (2024). A Glance on Gold Nanoparticle: An Emerging Theranostic Tool for Oncology. J. Drug Deliv. Sci. Technol..

[5] Gao Q., Zhang J., Gao J., Zhang Z., Zhu H., Wang D. (2021). Gold Nanoparticles in Cancer Theranostics. Front. Bioeng. Biotechnol..

[6] Pijeira M.S.O., Viltres H., Kozempel J., Sakmár M., Vlk M., İlem-Özdemir D., Ekinci M., Srinivasan S., Rajabzadeh A.R., Ricci-Junior E. (2022). Radiolabeled Nanomaterials for Biomedical Applications: Radiopharmacy in the Era of Nanotechnology. EJNMMI Radiopharm. Chem..

[7] Ladrière T., Faudemer J., Levigoureux E., Peyronnet D., Desmonts C., Vigne J. (2023). Safety and Therapeutic Optimization of Lutetium-177 Based Radiopharmaceuticals. Pharmaceutics.

[8] Jalilian A.R., Ocampo-García B., Pasanphan W., Sakr T.M., Melendez-Alafort L., Grasselli M., Lugao A.B., Yousefnia H., Dispenza C., Janib S.M. (2022). IAEA Contribution to Nanosized Targeted Radiopharmaceuticals for Drug Delivery. Pharmaceutics.

[9] Bentivoglio V., Nayak P., Varani M., Lauri C., Signore A. (2023). Methods for Radiolabeling Nanoparticles (Part 3): Therapeutic Use. Biomolecules.

[10] Luining W.I., Cysouw M.C.F., Meijer D., Hendrikse N.H., Boellaard R., Vis A.N., Oprea-Lager D.E. (2022). Targeting PSMA Revolutionizes the Role of Nuclear Medicine in Diagnosis and Treatment of Prostate Cancer. Cancers.

[11] Sun M., Niaz M.J., Niaz M.O., Tagawa S.T. (2021). Prostate-Specific Membrane Antigen (PSMA)-Targeted Radionuclide Therapies for Prostate Cancer. Curr. Oncol. Rep..

[12] Maes J., Gesquière S., De Spiegeleer A., Maes A., Van De Wiele C. (2024). Prostate-Specific Membrane Antigen Biology and Pathophysiology in Prostate Carcinoma, an Update: Potential Implications for Targeted Imaging and Therapy. Int. J. Mol. Sci..

[13] Jones W., Griffiths K., Barata P.C., Paller C.J. (2020). PSMA Theranostics: Review of the Current Status of PSMA-Targeted Imaging and Radioligand Therapy. Cancers.

[14] Naik M., Khan S.R., Lewington V., Challapalli A., Eccles A., Barwick T.D. (2024). Imaging and Therapy in Prostate Cancer Using Prostate Specific Membrane Antigen Radioligands. Br. J. Radiol..

[15] Ghosh A., Heston W.D.W. (2004). Tumor Target Prostate Specific Membrane Antigen (PSMA) and Its Regulation in Prostate Cancer. J. Cell. Biochem..

[16] Wright G.L., Haley C., Beckett M.L., Schellhammer P.F. (1995). Expression of Prostate-Specific Membrane Antigen in Normal, Benign, and Malignant Prostate Tissues. Urol. Oncol. Semin. Orig. Investig..

[17] O’Keefe D.S., Bacich D.J., Huang S.S., Heston W.D.W. (2018). A Perspective on the Evolving Story of PSMA Biology, PSMA-Based Imaging, and Endoradiotherapeutic Strategies. J. Nucl. Med..

[18] Apostolopoulou A., Salvanou E.-A., Chiotellis A., Pirmettis N.N., Pirmettis I.C., Xanthopoulos S., Koźmiński P., Bouziotis P. (2024). How Does the Concentration of Technetium-99m Radiolabeled Gold Nanoparticles Affect Their In Vivo Biodistribution?. Appl. Sci..

[19] Salvanou E.-A., Apostolopoulou A., Xanthopoulos S., Koelewijn S., Van Overeem P., Laurent G., Bazzi R., Denat F., Roux S., Bouziotis P. (2025). 161Terbium-Labeled Gold Nanoparticles as Nanoscale Brachytherapy Agents Against Breast Cancer. Materials.

[20] Salvanou E.-A., Kolokithas-Ntoukas A., Prokopiou D., Theodosiou M., Efthimiadou E., Koźmiński P., Xanthopoulos S., Avgoustakis K., Bouziotis P. (2024). 177Lu-Labeled Iron Oxide Nanoparticles Functionalized with Doxorubicin and Bevacizumab as Nanobrachytherapy Agents against Breast Cancer. Molecules.

[21] Felber M., Bauwens M., Mateos J.M., Imstepf S., Mottaghy F.M., Alberto R. (2015). ^99m^Tc Radiolabeling and Biological Evaluation of Nanoparticles Functionalized with a Versatile Coating Ligand. Chem. Eur. J..

[22] Morales-Avila E., Ferro-Flores G., Ocampo-García B.E., De León-Rodríguez L.M., Santos-Cuevas C.L., García-Becerra R., Medina L.A., Gómez-Oliván L. (2011). Multimeric System of ^99m^Tc-Labeled Gold Nanoparticles Conjugated to c[RGDfK(C)] for Molecular Imaging of Tumor α(v)β(3) Expression. Bioconjugate Chem..

[23] Salvanou E.-A., Stellas D., Tsoukalas C., Mavroidi B., Paravatou-Petsotas M., Kalogeropoulos N., Xanthopoulos S., Denat F., Laurent G., Bazzi R. (2020). A Proof-of-Concept Study on the Therapeutic Potential of Au Nanoparticles Radiolabeled with the Alpha-Emitter Actinium-225. Pharmaceutics.

[24] Karandish F., Haldar M.K., You S., Brooks A.E., Brooks B.D., Guo B., Choi Y., Mallik S. (2016). Prostate-Specific Membrane Antigen Targeted Polymersomes for Delivering Mocetinostat and Docetaxel to Prostate Cancer Cell Spheroids. ACS Omega.

[25] Fan D., Cao Y., Cao M., Wang Y., Cao Y., Gong T. (2023). Nanomedicine in Cancer Therapy. Signal Transduct. Target. Ther..

[26] Meher N., VanBrocklin H.F., Wilson D.M., Flavell R.R. (2023). PSMA-Targeted Nanotheranostics for Imaging and Radiotherapy of Prostate Cancer. Pharmaceuticals.

[27] Flores O., Santra S., Kaittanis C., Bassiouni R., Khaled A.S., Khaled A.R., Grimm J., Perez J.M. (2017). PSMA-Targeted Theranostic Nanocarrier for Prostate Cancer. Theranostics.

[28] Bajwa D.E., Salvanou E.-A., Theodosiou M., Koutsikou T.S., Efthimiadou E.K., Bouziotis P., Liolios C. (2023). Radiolabeled Iron Oxide Nanoparticles Functionalized with PSMA/BN Ligands for Dual-Targeting of Prostate Cancer. Front. Nucl. Med..

[29] Ponchelle M., Pruszyński M., Gravel E., Doris E. (2025). ^177^Lu-Gold Nanohybrids in Radiotherapeutic Approaches Against Cancer. Small Sci..

[30] Liolios C., Schäfer M., Haberkorn U., Eder M., Kopka K. (2016). Novel Bispecific PSMA/GRPr Targeting Radioligands with Optimized Pharmacokinetics for Improved PET Imaging of Prostate Cancer. Bioconjugate Chem..

[31] Benešová M., Bauder-Wüst U., Schäfer M., Klika K.D., Mier W., Haberkorn U., Kopka K., Eder M. (2016). Linker Modification Strategies To Control the Prostate-Specific Membrane Antigen (PSMA)-Targeting and Pharmacokinetic Properties of DOTA-Conjugated PSMA Inhibitors. J. Med. Chem..

[32] Schäfer M., Bauder-Wüst U., Leotta K., Zoller F., Mier W., Haberkorn U., Eisenhut M., Eder M. (2012). A Dimerized Urea-Based Inhibitor of the Prostate-Specific Membrane Antigen for 68Ga-PET Imaging of Prostate Cancer. EJNMMI Res..

[33] Apostolopoulou A., Chiotellis A., Salvanou E.-A., Makrypidi K., Tsoukalas C., Kapiris F., Paravatou-Petsotas M., Papadopoulos M., Pirmettis I.C., Koźmiński P. (2021). Synthesis and In Vitro Evaluation of Gold Nanoparticles Functionalized with Thiol Ligands for Robust Radiolabeling with ^99m^Tc. Nanomaterials.

[34] Turkevich J., Stevenson P.C., Hillier J. (1951). A Study of the Nucleation and Growth Processes in the Synthesis of Colloidal Gold. Discuss. Faraday Soc..

[35] Paparidis G., Akrivou M., Tsachouridou V., Shegani A., Vizirianakis I.S., Pirmettis I., Papadopoulos M.S., Papagiannopoulou D. (2018). Synthesis and Evaluation of ^99m^Tc/Re-Tricarbonyl Complexes of the Triphenylphosphonium Cation for Mitochondrial Targeting. Nucl. Med. Biol..

[36] Żelechowska-Matysiak K., Wawrowicz K., Wierzbicki M., Budlewski T., Bilewicz A., Majkowska-Pilip A. (2023). Doxorubicin- and Trastuzumab-Modified Gold Nanoparticles as Potential Multimodal Agents for Targeted Therapy of HER2+ Cancers. Molecules.

[37] Liu Y., Li H., Zhou H., Yuan H., Zhao Y., Yang Z., Tang S., Wu T., Wang L., Huang Z. (2025). In Vitro and In Vivo Study of Novel PSMA-Targeted Radioligands: Enhancing Tumor Uptake and Therapeutic Efficacy through Zwitterionization and Albumin-Binding Strategies. Mol. Pharm..

[38] Wawrowicz K., Majkowska-Pilip A., Gaweł D., Chajduk E., Pieńkowski T., Bilewicz A. (2021). Au@Pt Core-Shell Nanoparticle Bioconjugates for the Therapy of HER2+ Breast Cancer and Hepatocellular Carcinoma. Model Studies on the Applicability of 193mPt and 195mPt Radionuclides in Auger Electron Therapy. Molecules.

[39] Smith A.M., Marbella L.E., Johnston K.A., Hartmann M.J., Crawford S.E., Kozycz L.M., Seferos D.S., Millstone J.E. (2015). Quantitative Analysis of Thiolated Ligand Exchange on Gold Nanoparticles Monitored by ^1^H NMR Spectroscopy. Anal. Chem..

[40] Silva F., D’Onofrio A., Mendes C., Pinto C., Marques A., Campello M.P.C., Oliveira M.C., Raposinho P., Belchior A., Di Maria S. (2022). Radiolabeled Gold Nanoseeds Decorated with Substance P Peptides: Synthesis, Characterization and In Vitro Evaluation in Glioblastoma Cellular Models. Int. J. Mol. Sci..

[41] Felber M., Alberto R. (2015). ^99m^Tc Radiolabelling of Fe_3_O_4_–Au Core–Shell and Au–Fe_3_O_4_ Dumbbell-like Nanoparticles. Nanoscale.

[42] Yook S., Cai Z., Lu Y., Winnik M.A., Pignol J.-P., Reilly R.M. (2015). Radiation Nanomedicine for EGFR-Positive Breast Cancer: Panitumumab-Modified Gold Nanoparticles Complexed to the β-Particle-Emitter, ^177^Lu. Mol. Pharm..

[43] Chen M.-W., Huang Y.-R., Lo W.-L., Lee S.-Y., Lo S.-N., Wang S.-M., Chang K.-W. (2025). PSMA-Targeted Radiolabeled Peptide for Imaging and Therapy in Prostate Cancer: Preclinical Evaluation of Biodistribution and Therapeutic Efficacy. Int. J. Mol. Sci..

[44] Van Zundert I., Fortuni B., Rocha S. (2020). From 2D to 3D Cancer Cell Models—The Enigmas of Drug Delivery Research. Nanomaterials.

[45] Lee S.-Y., Koo I.-S., Hwang H.J., Lee D.W. (2023). In Vitro Three-Dimensional (3D) Cell Culture Tools for Spheroid and Organoid Models. SLAS Discov..

[46] Park S.Y., Hong H.J., Lee H.J. (2023). Fabrication of Cell Spheroids for 3D Cell Culture and Biomedical Applications. BioChip J..

[47] Arora S., Singh S., Mittal A., Desai N., Khatri D.K., Gugulothu D., Lather V., Pandita D., Vora L.K. (2024). Spheroids in Cancer Research: Recent Advances and Opportunities. J. Drug Deliv. Sci. Technol..

[48] Anisimov R.A., Gorin D.A., Abalymov A.A. (2022). 3D Cell Spheroids as a Tool for Evaluating the Effectiveness of Carbon Nanotubes as a Drug Delivery and Photothermal Therapy Agents. C.

[49] Živković Z., Opačak-Bernardi T. (2025). An Overview on Spheroid and Organoid Models in Applied Studies. Sci.

[50] Blondeel E., Peirsman A., Vermeulen S., Piccinini F., De Vuyst F., Estêvão D., Al-Jamei S., Bedeschi M., Castellani G., Cruz T. (2025). The Spheroid Light Microscopy Image Atlas for Morphometrical Analysis of Three-Dimensional Cell Cultures. Sci. Data.

[51] Taylor R.M., Severns V., Brown D.C., Bisoffi M., Sillerud L.O. (2012). Prostate Cancer Targeting Motifs: Expression of α_ν_β_3_, Neurotensin Receptor 1, Prostate Specific Membrane Antigen, and Prostate Stem Cell Antigen in Human Prostate Cancer Cell Lines and Xenografts. Prostate.

[52] Laidler P., Dulińska J., Lekka M., Lekki J. (2005). Expression of Prostate Specific Membrane Antigen in Androgen-Independent Prostate Cancer Cell Line PC-3. Arch. Biochem. Biophys..

[53] Wang L., Tang L., Liu Y., Wu H., Liu Z., Li J., Pan Y., Akkaya E.U. (2022). Prostate-Specific Membrane Antigen (PSMA) Targeted Singlet Oxygen Delivery *via* Endoperoxide Tethered Ligands. Chem. Commun..

[54] Senrung A., Lalwani S., Janjua D., Tripathi T., Kaur J., Ghuratia N., Aggarwal N., Chhokar A., Yadav J., Chaudhary A. (2023). 3D Tumor Spheroids: Morphological Alterations a Yardstick to Anti-Cancer Drug Response. Vitr. Model..

[55] Liolios C., Patsis C., Lambrinidis G., Tzortzini E., Roscher M., Bauder-Wüst U., Kolocouris A., Kopka K. (2022). Investigation of Tumor Cells and Receptor-Ligand Simulation Models for the Development of PET Imaging Probes Targeting PSMA and GRPR and a Possible Crosstalk between the Two Receptors. Mol. Pharm..

[56] Matthias J., Engelhardt J., Schäfer M., Bauder-Wüst U., Meyer P.T., Haberkorn U., Eder M., Kopka K., Hell S.W., Eder A.-C. (2021). Cytoplasmic Localization of Prostate-Specific Membrane Antigen Inhibitors May Confer Advantages for Targeted Cancer Therapies. Cancer Res..

[57] Rajasekaran S.A., Anilkumar G., Oshima E., Bowie J.U., Liu H., Heston W., Bander N.H., Rajasekaran A.K. (2003). A Novel Cytoplasmic Tail MXXXL Motif Mediates the Internalization of Prostate-Specific Membrane Antigen. MBoC.

[58] Liu H., Rajasekaran A.K., Moy P. (1998). Constitutive and Antibody-Induced Internalization of Prostate-Specific Membrane Antigen. Cancer Res..

