# Supplementary material for: Lutetium-177 Radiolabeled Gold Nanoparticles for Prostate Cancer Theranostics
Source: Nanomaterials (Basel). 2026 Apr 4;16(7):441. doi: 10.3390/nano16070441 (PMC13074626; doi:10.3390/nano16070441)
Supplement: Supplementary file 1 [file nanomaterials-16-00441-s001.zip › nanomaterials-4202276-supplementary.pdf]

nanomaterials

Supplementary Information

Lutetium-177 Radiolabeled Gold Nanoparticles for Prostate Cancer Theranostics

Adamantia Apostolopoulou[1,2], Evangelia-Alexandra Salvanou[1], Christos Liolios[1,3], Stavros Xanthopoulos[1], Przemysław Koźmiński[4] and Penelope Bouziotis[1*]

[1] Radiochemical Studies Laboratory, Institute of Nuclear & Radiological Sciences & Technology, Energy & Safety, National Center for Scientific Research "Demokritos", 15341 Agia Paraskevi, Athens, Greece; a.apostolopoulou@rrp.demokritos.gr (A.A.); salvanou@rrp.demokritos.gr (E-A.S.); cliolios@pharm.uoa.gr (C.L); staxan@rrp.demokritos.gr (S.X.); bouzioti@rrp.demokritos.gr (P.B.)

[2] Department of Medicine, National and Kapodistrian University of Athens, Mikras Asias 75, 11527, Athens, Greece; a.apostolopoulou@rrp.demokritos.gr (A.A.)

[3] Department of Pharmacy, National and Kapodistrian University of Athens, Panepistimiopolis Zographou 15771 Athens, Greece; cliolios@pharm.uoa.gr (C. L)

[4] Centre of Radiochemistry and Nuclear Chemistry, Institute of Nuclear Chemistry and Technology, Dorodna 16 Str., 03-195 Warsaw, Poland; p.kozminski@ichtj.waw.pl (P.K.)

* Correspondence: bouzioti@rrp.demokritos.gr; +30-2106503687 (P.B.)

Table of Contents

1. Synthesis and characterization of the PSMA-targeting molecule
2. The RP-HPLC and ESI-MS analysis of pure (8) PSMA-SH
3. Radiochemical Yield and *in vitro* stability studies
4. Statistical analysis on LNCaP and PC3 spheroids

1. Synthesis and characterization of the PSMA-targeting molecule

The synthesis of the PSMA-SH derivative started with **(1)** (PSMA-Alloc on a 2-CTC resin). A quantity of resin (0.180 g) was added in a syringe reactor and swelled (2 x 5.0 mL DCM and 3 x 5.0 mL dry DCM). PSMA-Alloc **(1)** was synthesized on the resin (2-Chlorotritylresin (2-CTC) (0.3 mmol, substitution capacity 1.22 mmol/g, 100–200 MESH) according to previously published methods.

Alloc Deprotection (2): The alloc group was removed according to previously known methodS. Briefly the resin reacted with 2 x solution A (Solution A: 25 mg Pd (tetrakis(triphenylphosphine)palladium(0) in 2.5 mL dry DCM and 0.4 mL morpholine). The syringe was covered in aluminum foil because Pd is photosensitive and left under gentle stirring for 1 h at RT. The process was repeated with fresh Solution A. Finally, the resin was washed with 1% DIPEA in DMF (300 μL DIPEA in 30 mL DMF), sodium di-ethyldithiocarbamate (0.450 g in 30 mL DMF) and DMF. The resin was checked with a Kaiser test for the efficiency of Alloc deprotection (positive test) before proceeding to the next coupling step.

Synthesis of **3-7***:* For the amino acid (a.a.) couplings the Oxyma pure/DIC protocol was used in DMF. Briefly, 4 equiv. of each a.a., (Fmoc-Phe-OH (465 mg), synthesis of **3**, and Fmoc-Cys(Trt)-OH (702 mg, synthesis of **5**), were dissolved in DMF (0.60 mL) and mixed with oxyma pure (170.5 mg, 0.30 mL DMF) (0 °C, 4 min). Thereafter, DIC (0.180 mL) was added to the vial and (0 °C, 5 min) and the mixture was added to reactor syringe and left to react for 1 h at RT, under gentle stirring. Each coupling was followed by resin washing (DMF, 5 x 1 min). and a Kaiser test (negative Kaiser = efficient coupling). After each coupling the Fmoc group was removed with 20% 4-methyl-piperidine (2 x 10 min) and washed with DMF (10 x 1 min), synthesis of **4** and **6** respectively.

*Cleavage from the Resin (synthesis of **7-8**):* Before peptide cleavage from the resin, the free alpha -NH2 group of 6 was capped with DMF: Ac2O: DIPEA (1:1:1), (V = 0.9 mL) resulting in 7 (showing a negative Kaiser test). Then, the resin was washed (5 mL/g resin) with DMF (3 x 1 min), DCM (3 x 1 min), and petroleum ether (2 x 1 min) and dried under vacuum. Then, the following cleavage mixture was applied: 94% TFA, 2.5% water, 2.5% EDT and 1% TIS (v/v/v/v) (3 h under gentle stirring, RT). The peptide was precipitated in cold (0 °C) diethyl ether and the mixture was centrifuged at 3300 rpm for 15 min. The supernatant was removed and the pellet was dissolved in MeOH and purified with RP-HPLC (gradient system: 10% B to 80% B at 13 min, solvent A: H_2O/0.1% TFA and solvent B: ACN/0.1% TFA, flow rate = 2 mL/min using a semi-prep C18 column). The main peak was collected and product **8** was identified by ESI-MS.

2. The RP-HPLC and ESI-MS analysis of pure (8) PSMA-SH

The pure product 8 was analyzed with RP – HPLC (Figure S1) and identified with ESI-MS (S.I) (Figure S2)

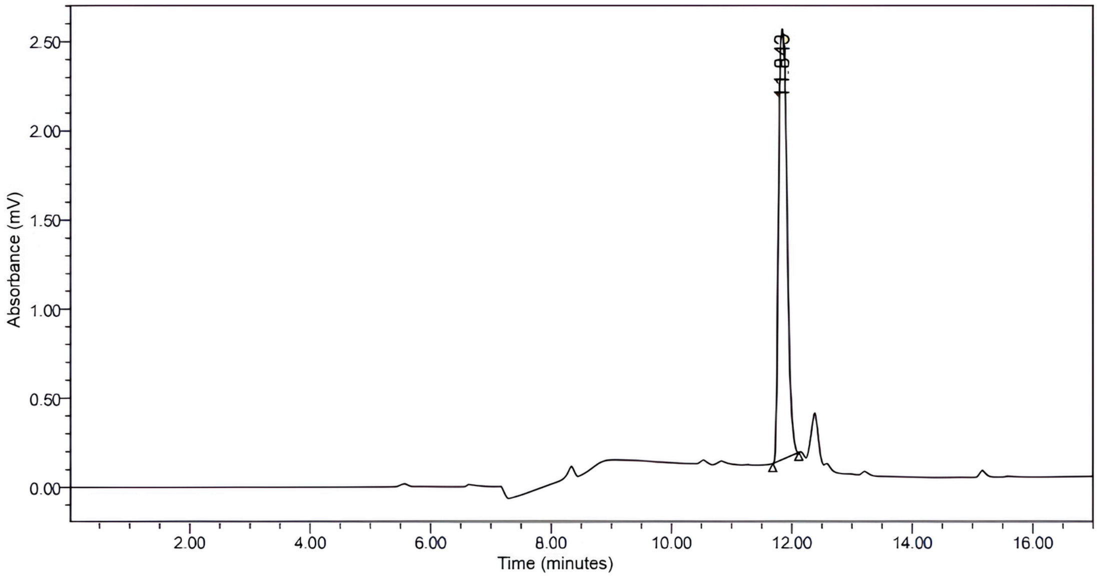

Figure S1. HPLC graph representing the pure synthesized PSMA-SH on the UV (254 nm) applying a gradient system from 10% B to 80% B at 13 min and from 80% B at 15 min to 10% B at 17 min, where solvent A was H_2O/0.1% TFA and solvent B was ACN/0.1% TFA, at a flow rate of 2 mL/min using a semi-prep C18 column.

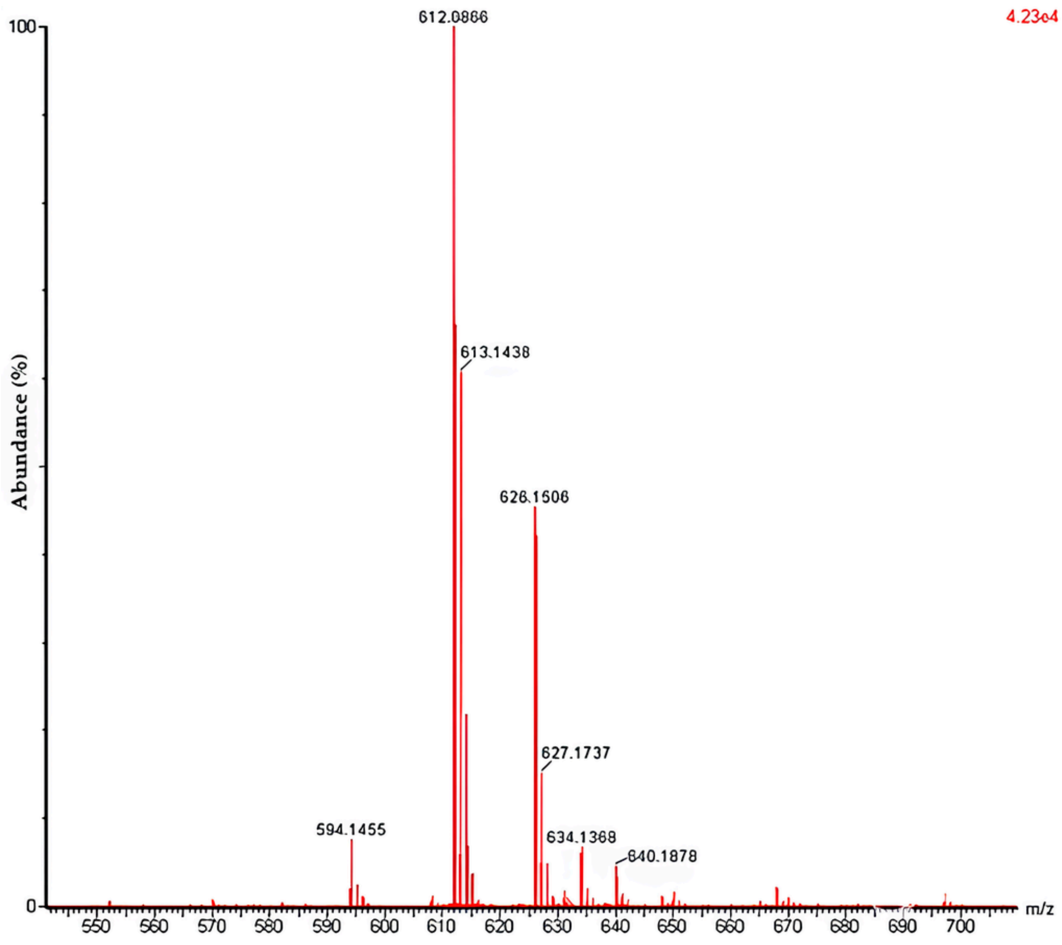

Figure S2. Mass Spectrometry graph for the synthesized product PSMA-SH, MW: 611.6670, m/z (M+H) experimental: 612.0866, m/z (M+2H), 613.1438 (60%).

3. Radiochemical Yield and in vitro stability studies

For experimental details see also 2.5. Radiolabeling of AuNPs with Lutetium-177 and 2.6. In vitro stability studies. The results are presented in Tables S1 and S2.

Table S1. Radiochemical yield and stability data of [^{177}Lu]Lu-AuNPs-TADOTAGA at RT (Bench stability), in the presence of human serum, PBS and DTPA for up to 15 days.

	RY (%)	RT (%)	Human Serum (%)	PBS (%)	DTPA (%)
	98.55 ± 0.63				
Day 1		96.48 ± 1.63	96.54 ± 1.10	96.93 ± 0.82	93.75 ± 3.21
Day 2		96.59 ± 1.56	95.91 ± 0.87	96.90 ± 1.32	95.22 ± 0.50
Day 4		95.25 ± 1.37	95.07 ± 2.45	96.31 ± 0.95	91.79 ± 4.70
Day 7		94.96 ± 1.10	96.07 ± 2.06	95.37 ± 1.63	93.58 ± 4.57
Day 9		92.39 ± 5.08	92.73 ± 5.85	94.16 ± 2.22	88.35 ± 6.53
Day 15		95.22 ± 4.16	80.59 ± 6.32	92.02 ± 2.60	85.96 ± 3.57

Table S2. Radiochemical yield and stability data of [^{177}Lu]Lu-AuNPs-TADOTAGA-PSMA at RT (Bench stability), in the presence of human serum, PBS and DTPA for up to 15 days.

	RY (%)	RT (%)	Human Serum (%)	PBS (%)	DTPA (%)
	97.15 ± 1.69				
Day 1		95.97 ± 0.86	97.10 ± 0.86	97.36 ± 0.67	93.93 ± 2.51
Day 2		96.60 ± 1.67	95.33 ± 3.65	95.81 ± 3.23	94.85 ± 1.41
Day 4		95.78 ± 2.18	96.66 ± 0.89	93.47 ± 2.20	92.07 ± 0.52
Day 7		92.04 ± 1.34	91.31 ± 3.32	92.56 ± 1.81	86.06 ± 6.51
Day 9		89.92 ± 2.29	87.91 ± 2.85	92.75 ± 2.39	83.91 ± 1.70
Day 15		90.45 ± 4.54	86.92 ± 3.59	89.14 ± 2.91	85.96 ± 0.67

4. Statistical analysis on LNCaP and PC3 spheroids

For experimental details see also 2.8.2 Cytotoxicity studies on 3D cell spheroids. The results are presented in Figures S3 and S4.

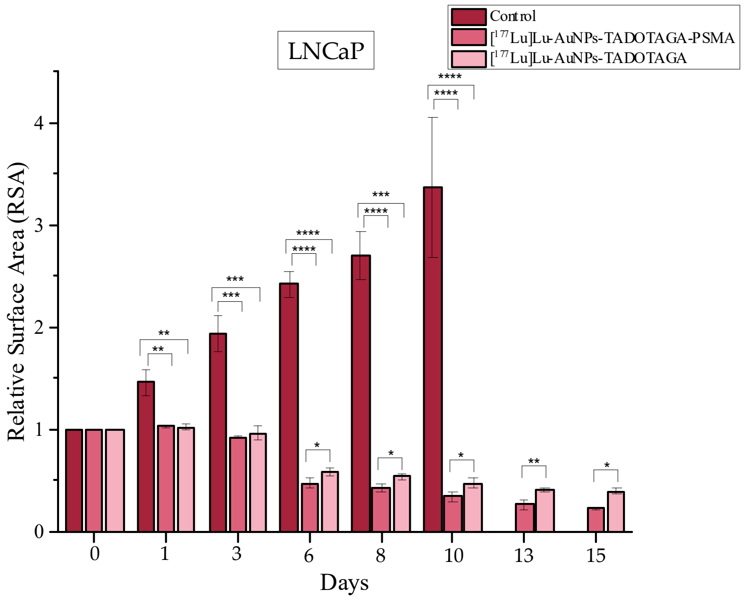

Figure S3. LNCaP spheroids treated with [^{177}Lu]Lu-AuNPs-TADOTAGA-PSMA and [^{177}Lu]Lu-AuNPs-TADOTAGA or non-treated (Control group). (* $p < 0.05$, ** $p < 0.01$, *** $p < 0.001$, **** $p < 0.0001$; absence of asterisks indicates a non-significant statistical difference). Values represent the mean ± SD (n = 3).

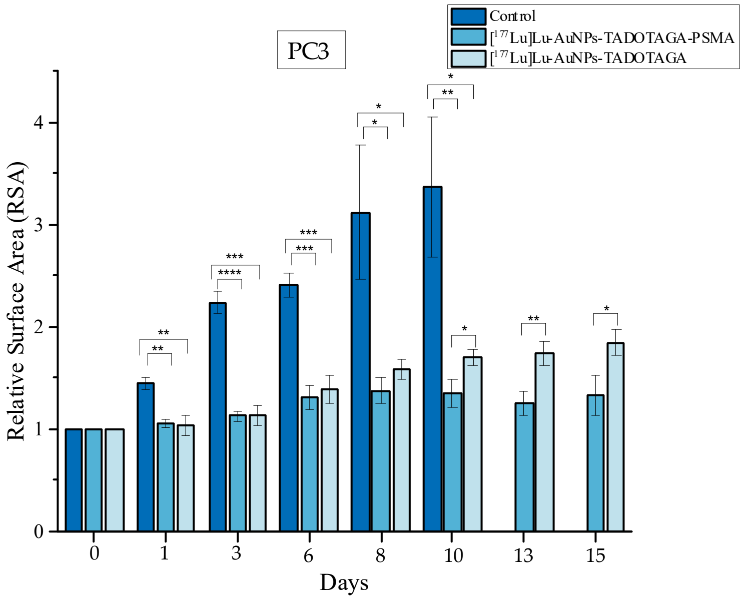

Figure S4. PC3 spheroids treated with [^{177}Lu]Lu-AuNPs-TADOTAGA-PSMA and [^{177}Lu]Lu-AuNPs-TADOTAGA or non-treated (Control group). (* $p < 0.05$, ** $p < 0.01$, *** $p < 0.001$, **** $p < 0.0001$; absence of asterisks indicates a non-significant statistical difference). Values represent the mean ± SD (n = 3).
